# Role of Speckle Tracking Echocardiography in Dilated Cardiomyopathy: A Review

**DOI:** 10.7759/cureus.1372

**Published:** 2017-06-20

**Authors:** Ghulam Murtaza, Hafeez Ul Hasan Virk, Muhammad Khalid, Zia Rahman, Puja Sitwala, Jeffrey Schoondyke, Kais Al-Balbissi

**Affiliations:** 1 Department of Internal Medicine, East Tennessee State University; 2 Department of Medicine, St. Luke’s-Roosevelt Hospital Center, Icahn School of Medicine at Mount Sinai, New York City, New York; 3 Department of Internal Medicine, Division of Cardiology, East Tennessee State University; 4 Division of Cardiology, East Tennessee State University

**Keywords:** speckle, tracking, cardiomyopathy

## Abstract

Dilated cardiomyopathy (DCM) is an important cause of the heart failure. Timely diagnosis and optimal management decrease morbidity and mortality in heart failure patients. Although transthoracic echocardiography is used as the diagnostic test of choice in these patients, new modalities like speckle tracking echocardiography (STE) have promising results in diagnosing these patients in the earlier course of the disease. Advancements in cardiac imaging are expected as more clinical studies on the role of STE in different cardiac diseases that emerge. In this review article, we will discuss the basics of STE and its role in diagnosing DCM.

## Introduction and background

Heart failure (HF) is a growing public health concern, costing the United States (US) approximately 31 billion dollars annually [1]. Cardiomyopathies are among the important causes of HF and dilated cardiomyopathy (DCM) is the most common type responsible for about 10,000 deaths and 46,000 hospitalizations in the US every year [2]. Its incidence is five to eight cases per 100,000 people annually, with a prevalence of 36 per 100,000 people [3]. DCM is a final common pathway of most of the cardiac diseases and is characterized by dilatation and impaired contraction or systolic dysfunction of the left and sometimes right ventricle [3-6]. Most commonly, it presents with the typical symptoms of left ventricular (LV) heart failure (i.e., dyspnea on exertion, impaired exercise tolerance, orthopnea, paroxysmal nocturnal dyspnea, and peripheral edema). Less often, DCM may present with arrhythmias, conduction disturbances, thromboembolic complications or sudden death [7]. DCM sequelae include cardiac remodeling, HF with reduced ejection fraction (EF), and in some cases, HF with preserved EF. At present, the classification of DCM excludes cases in which coronary artery disease (CAD), hypertension, valvular diseases, and congenital heart diseases have contributed to this phenotype [8]. The diagnosis of DCM involves many diagnostic techniques, but transthoracic echocardiography remains the cornerstone of the investigative pathway. Speckle tracking echocardiography (STE) has emerged as a novel technology to detect abnormalities in the heart to further refine the diagnosis and management of cardiac diseases. This review will shed some light on the mechanistic insights of DCM for a better understanding of its pathophysiology and elucidate some new imaging biomarkers through STE. Some of the common etiologies of dilated cardiomyopathy are shown in Table 1.

**Table 1:** Etiologies of dilated cardiomyopathies

Etiology	Examples
Valvular	Volume overload conditions (e.g., mitral regurgitation, aortic regurgitation)
Familial	25% of cases, autosomal dominant, X-linked recessive, mitochondrial
Metabolic	Nutritional deficiencies, diabetes mellitus, hypophosphatemia, hypocalcemia, thiamine deficiency, selenium deficiency, carnitine deficiency
Endocrinopathies	Hypothyroidism, thyrotoxicosis, pheochromocytoma, Cushing disease
Infectious	Viral ( human immunodeficiency virus (HIV), parvoB19, human herpesvirus (HHV-6), bacterial, fungal, mycobacterial, rickettsia
Toxins	Alcohol abuse, cocaine abuse
Medications	Antiretrovirals, lithium, phenothiazines, anthracyclines, trastuzumab
Autoimmune	Systemic lupus erythematosus, sarcoidosis, amyloidosis, idiopathic giant cell myocarditis
Hematological related	Sickle cell disease, chronic anemia, thalassemia
Muscle dystrophies	Duchenne, Becker disease
	Tachycardia-induced cardiomyopathy (supraventricular and ventricular)
	Peripartum cardiomyopathy (last month to five months post-partum, increased risk with multiparity)
Takotsubo cardiomyopathy	Stress-induced cardiomyopathy or broken heart syndrome: characterized by apical ballooning and usually resolves over weeks
Idiopathic	Nearly 25% of the cases after excluding all recognizable etiologies

## Review

### Genetic and mechanics of dilated cardiomyopathy

Regardless of the cause, both genetic and environmental factors (e.g., hemodynamics) play a role in left ventricular (LV) dilatation, systolic dysfunction, myocyte death, and myocardial fibrosis leading to remodeling. Genetically, mutations in the genes encoding structural and contractile proteins result in functional changes. Mutations in the Beta (β)-myosin heavy chain gene impairs motor function in DCM [9-10] and mutations in genes for thin-filament regulatory proteins reduce the affinity of troponin for calcium [11-12]; hence, the generation of force is not achieved.

From a mechanical perspective, patients with DCM have an increased LV mass and volume due to the thinning and stretching of the LV wall [13]. These changes lead to reduced strain in all directions (e.g., longitudinal, radial, and circumferential) [14-16]. As a result of these changes in the mechanics of the heart’s pumping function, fewer symptoms are associated with higher cardiac strain [17]. Similarly, dyssynchrony in circumferential and longitudinal strain predicts rapid HF progression. Likewise, LV rotation is reduced at the base and apex, which mitigates LV twisting [15-16,18] and untwisting velocities [19]. The paradoxical reversal of LV rotation direction may be perceived with the LV base establishing counterclockwise rotation and the apex demonstrating clockwise rotation [18-21].

### Diagnostic modalities

There is no single test to diagnose DCM, although systolic dysfunction/failure can be assessed at bedside based on clinical signs and symptoms. Echocardiography will show the dilation of the heart’s chambers with reduced ejection fraction (EF) along with akinesia or a thinning of the ventricular walls. Cardiac magnetic resonance imaging (MRI) is another very helpful diagnostic study if suspicion of myocarditis or infiltrative cardiomyopathy is high. Cardiac MRI is 76% sensitive and 96% specific for these etiologies [22]. Stress tests and coronary angiography are other options if ischemia is suspected or CAD is high to prevent hibernating myocardium over time. Myocardial biopsy is another option if an acute hemodynamic compromise is noted and can rule out giant cell myocarditis. However, myocardial biopsy carries a high rate of false negatives. Diagnosing a patient with DCM early in the disease is prudent as cardiomyopathy caused by reversible factors or diseases can be addressed to avoid further comorbidities. DCM is diagnosed based on the presence of dilatation with resultant impaired contractility/systolic function of one or both ventricles (e.g., left ventricular ejection fraction (LVEF) < 40% to 50% or fractional shortening < 25%) [2,4]. After excluding primary or secondary causes of cardiomyopathy such as valvular heart disease, myocarditis or significant CAD, idiopathic DCM should be considered [2-3].

### Role of transthoracic echocardiography in dilated cardiomyopathy 

The conventional two-dimensional (2D) transthoracic echocardiographic (TTE) findings in DCM are LV spherical dilatation, normal/reduced wall thickness, and reduced inward endocardial systolic motion. Systolic indices which include LV fractional shortening, fractional area change, and EF are reduced mainly because of four-chamber cardiac enlargement. Due to its inherent advantages of economy, safety, and portability, conventional TTE plays an important role in the diagnosis of DCM. However, there is growing need for novel emerging imaging modalities due to DCM’s complex pathophysiology, high incidence and prevalence, and extremely high economic burden combined with a lack of diagnostic yield from conventional echocardiography and suboptimal elucidation from mechanistic insights.

### Basics of speckle tracking echocardiography

The myocardial strain is a relatively new metric for heart function and it involves measuring the change of shape or deformation of the heart chambers. The strain has many parameters as shown in Table 2.

**Table 2:** Various parameters of strain used in speckle tracking echocardiography

Variable	Description
Strain	Change in length relative to original length (L-Lo/Lo)
Strain rate	Rate of change of strain or change in strain over time showing systolic and diastolic phenomenon
Longitudinal Strain	Strain along the long axis of the left ventricle
Radial Strain	Thickening or thinning of myocardium
Circumferential strain	Strain along the circumference of left ventricle
Rotation	Direction of movement of base or apex of the heart
Twist	Difference in rotation of apex and base of the heart
Torsion	Twist divided by length of left ventricle

While myocardial strain is measured by many techniques, the two most notable methods are Doppler strain echocardiography (DSE) and STE. DSE is dependent on the angle of insonation and has a low lateral resolution. DSE is also fraught with tethering artifacts and requires mostly high frame rates (i.e., 120 to 160 frames per second- FPS) or higher.

STE is robust, reproducible, and independent of the degree of insonation, and STE needs relatively low frame rates (i.e., 60 to 90 FPS). The underlying physical principles are slightly complex but very interesting.

When sound waves come in contact with different tissue interfaces, they are reflected, refracted, absorbed or scattered. Very small wavelengths create small grainy densities or dots on the echo image; these dots are called “speckles.” The speckles are formed by the constructive and destructive interference of subwavelength sound waves. Normally, when a small wave coheres with another wave, (i.e., its crest falls on the other wave’s crest, and its trough falls on the trough of the other wave), we get an augmented or enhanced wave which gives a bright signal on the echo and a white dot appears. When waves decohere (i.e., the crest of one wave falls on the trough of another), they nullify or cancel each other, and we get dark spots or dark grains on the echo image.

**Figure 1:**
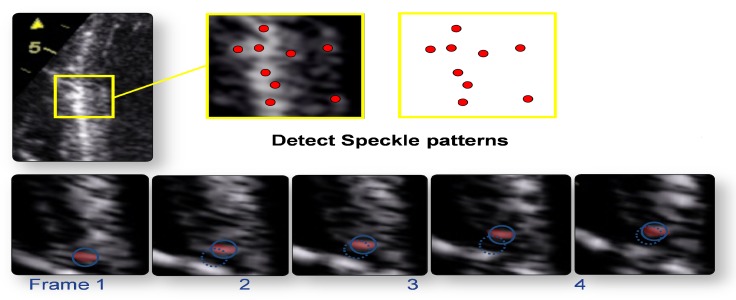
Figure showing the detection of speckle pattern of consecutive two dimensional (2D) frames. Myocardial motion characterization by natural acoustic “tagging"

Speckles are integrated into a functional unit called Kernels, which constitute a sort of ultrasound fingerprint that can be tracked by software during the entire cardiac cycle to produce a routine 2D grayscale image. Through this imaging system, we can calculate displacement, the rate of displacement, deformation, and the rate of deformation of the selected myocardial segment and LV rotation. It also provides an in-depth and noninvasive evaluation of myocardial dynamics during the systolic and diastolic phases of the cardiac cycle. It can also quantify rotational movements such as rotation, twisting, and torsion. The clinical application of STE has diagnostic, therapeutic, and prognostic value (Figure 1).

### Role of speckle tracking echocardiography in dilated cardiomyopathy

**Figure 2:**
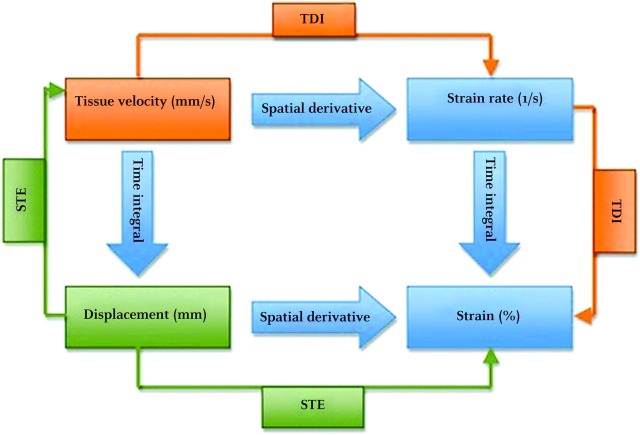
Figure showing mathematical relationship between strain and strain rate Abbreviation: Speckled tracking echocardiography (STE); Tissue doppler imaging (TDI)

STE is an emerging modality which has proven its mettle in the research arena and is now knocking at the doors of clinical practice. Speckle tracking derived strain (a measure of deformation or change in shape) is a load-independent measure of cardiac function. As opposed to conventional measures like EF, it can give a measure of both global and regional cardiac function. STE has emerged as an important diagnostic modality in cardiac diagnostic imaging [22-36]. In this modality, speckles (myocardial backscatters) are tracked frame by frame throughout the cardiac cycle and the deformation of indices are calculated (Figure 2).

**Figure 3:**
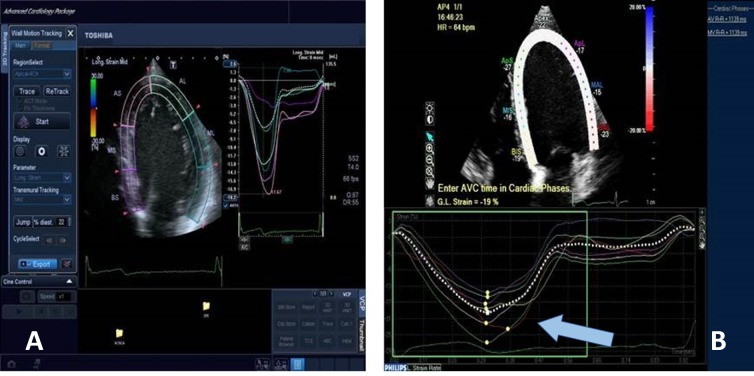
Figure showing the assessment of global longitudinal strain by speckled tracking echocardiography A) The graph represents strain rate (%) over time (in seconds) in a healthy individual. B) The graph represents strain rate (%) over time (in seconds) in a patient with dilated cardiomyopathy. Note: The decrease in global longitudinal strain in the patient with dilated cardiomyopathy (is indicated by the blue arrow)

In addition, the base and apex of the heart rotate in opposite directions to cause a twisting or writhing/rinsing movement which comprises 70% of the EF during systole and creates a suction pressure during diastole. Twist when indexed to the length of LV is called torsion [36-38] (Figure 3).

STE has been extensively used to assess a variety of cardiovascular pathologies. STE is superior to DSE because STE is not dependent on the angle of insonation, and tethering artifacts are not a major issue in STE. Compared to DSE, STE frame rate requirements are lower, computation time is shorter, and results are more reproducible [39].

LV subendocardial longitudinal functions the earliest factor to get deranged in DCM. Cristina, et al. demonstrated that LV longitudinal global strain is markedly decreased in DCM when compared with healthy controls [40]. The caveat of the study was patients who were not age-, gender- or body mass index (BMI) matched. The DCM patients were predominantly older males which may have affected the data. The authors explained that despite this non-matched cohort, the strain data are in synchrony with conventional echocardiography features. While this makes logical sense, a closer look at table 2 and figure 3 shows that global longitudinal strain (GLS) has a highly significant correlation (r = 0.61, P = .0001). In Table 2, the sample size is slightly biased for the DCM group with fewer healthy females, and BMI was not taken into consideration although body surface area was matched across both groups. This is acceptable in moderately sized cohorts as we sometimes do not have the luxury of matched groups.

Matsumoto, et al. used novel real-time 3-dimensional (3D) echocardiographic-based STE to describe 3D twist, rotation, and torsion mechanics in ischemic DCM with normal and prolonged QRS and compared these two cohorts with healthy cohorts [30]. They showed that not only is rotational mechanism disturbed in ischemic DCM, but it also leads to electrical dyssynchrony or vice versa. Interestingly, further supporting this hypothesis, twist and torsion moved towards normalization after cardiac resynchronization therapy (CRT). The authors provided some good mechanistic explanations for these findings. According to the proposed hypothesis, LV myocardial fibers are in cross-fiber arrangements at an angle of near 20 radians. When a muscle fiber unit shortens in one direction, it lengthens or shortens in two other directions. According to basic experimental studies, the strain generated in the longitudinal direction is -8% and maximized during thickening (i.e., radial strain), which is 32%, on average. Another interesting point raised by Matsumoto, et al. discusses the relationship of LV torsion with myocardial oxygen consumption and blood flow to the heart muscles [30]. Blood flow increases with increasing torsion, and increased twist leads to decreased myocardial oxygen consumption which might be due to a more energetically stable state of the heart. They reiterated the well-known fact that LV twist and torsion are the prime mechanisms in systole and diastole, creating suction and relaxation to complete the heart cycle. LV systolic twist and torsion accounts for approximately 60% to 70% of EF due to the high cardiac pressure gradient.

### Left ventricular torsion

The LV apex and base rotate in opposite directions because the endocardial and epicardial fibers are cross-woven at an angle, and epicardial fibers are longer than endocardial fibers. This cross-woven arrangement leads to the opposite rotation of the apex and base and the twisting of LV. This twist generates 60% to 70% of the ejection force. When this twist is indexed to LV length, we get torsion.

Similarly, untwisting occurs during isovolumic relaxation of the heart and leads to relaxation of LV and generates the necessary suction pressure for LV filling.

LV torsion mechanics have an important role in the physiology and pathophysiology of DCM. LV apical systolic rotation provides the main thrust for the ejection of blood while diastolic apical rotation causes suction. In DCM, there is a vicious cycle of reversing apical LV rotation which leads to LV remodeling, which further feeds into a negative loop.

In the early stages of DCM, relaxation is impaired and is the sole abnormality. In later stages, raised left atrial (LA) pressure becomes the main driver of EF.

Transverse motion of the apex is an emerging index associated with radial dyssynchrony and can be used to predict the response to CRT [41].

### Left atrial strain

**Figure 4:**
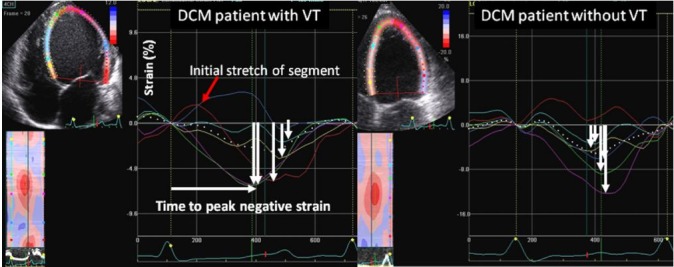
Figure showing longitudinal myocardial deformation in patient with dilated cardiomyopathy

Left atrial (LA) global longitudinal strain (GLS) is reduced in patients with DCM and reservoir and booster pump functions are reliable indices of LV filling pressure in DCM [42] (Figure 4).

Speckle tracking studies show DCM has biventricular involvement. The biventricular contractile reserve also offers a better predictive potential in DCM. Since DCM involves all age groups, a study in pediatric population highlighted LV radial dysfunction in DCM and radial dyssynchrony as the responsible mechanism [15]. However, the deformation abnormalities are not restricted to the radial strain. Rather, longitudinal, radial, circumferential strain, strain rate, twist, and torsion are also affected in DCM [43]. LA involvement in DCM has also been well documented [44].

Haugaa, et al. explored LV GLS in a selection of candidates for CRT and the results were quite favorable [29]. Compared with the cutoff of an EF < 35% which had both false positive and false negative results, even the electrophysiological criterion of QRS duration combined with EF could not supersede the prognosis, appropriate patient selection, and favorable outcomes. The study also demonstrated the significance of mechanical dyssynchrony as defined by time to peak longitudinal strain (T2P) in all 16 segments of the heart. The results showed that mechanical dispersion as evidenced by T2P > 72 ms resulted in decreased survival and worse prognosis. In brief, this study introduced mechanical dispersion and GLS as strong predictors of arrhythmogenic events in DCM.

## Conclusions

STE is an emerging modality which has shown great promise in the evaluation of dilated cardiomyopathies. We have been able to diagnose premature DCM and unravel the mechanistic insights. Therapeutic targets and prognostic landmarks have been identified with the help of STE. It has shown immense potential for use as surrogate endpoints in DCM clinical trials. Mechanical dyssynchrony has been very well studied, and in the future, we may be able to better select the DCM patient population with biventricular failure for cardiac resynchronization therapy (CRT) placement. DCM and STE together can contribute to better patient outcomes.
